# GLP-1 Receptor Signaling and Oral Dysfunction: A Narrative Review on the Mechanistic Basis of Semaglutide-Related Oral Adverse Effects

**DOI:** 10.3390/biology14121650

**Published:** 2025-11-23

**Authors:** Milena Barać, Jelena Roganović

**Affiliations:** Department of Pharmacology in Dentistry, School of Dental Medicine, University of Belgrade, 1 Dr Subotić Street, Number 1, 11000 Belgrade, Serbia; milena.barac@stomf.bg.ac.rs

**Keywords:** glucagon-like peptide 1 receptor agonists, semaglutide, salivary glands, xerostomia, cyclic AMP, beta-arrestins

## Abstract

This review addresses the growing concern of oral side effects, particularly dry mouth, associated with semaglutide, a glucagon-like peptide-1 receptor agonist (GLP-1RA) widely used for diabetes and obesity. A literature search of PubMed/MEDLINE, Scopus, Web of Science, and Google Scholar (March–September 2025) identified studies on GLP-1 receptor signaling, semaglutide pharmacology, salivary gland biology, biased agonism, β-arrestin, and cAMP pathways, and reported oral adverse effects. Of 183 records screened, 78 met inclusion criteria and were narratively synthesized across 5 mechanistic domains linking the molecular mechanisms that may underly semaglutide-induced alteration in salivary function by exploring GLP-1 receptor (GLP-1R) expression and signaling in salivary glands. The available literature data shows that different GLP-1 receptor agonists exhibit distinct patterns of GLP-1R activation, engaging the cAMP- and β-arrestin–dependent pathways to varying extents, which may thus differentially regulate exocytosis and cellular protection. Furthermore, semaglutide’s strong albumin binding leads to prolonged receptor activation, and may disturb the rhythmic calcium and cAMP cross-talk essential for normal salivary secretion. Persistent stimulation may cause receptor desensitization, β-arrestin–mediated internalization, and reduced gland responsiveness. Clinical pharmacovigilance data indicate disproportionality signals, suggesting that semaglutide may be reported more frequently with oral side effects compared with other GLP-1 receptor agonists, although spontaneous-report databases cannot confirm causality. These insights underscore the need for patient counseling, preventive oral care, and further studies on receptor signaling bias, contributing to personalized approach when using GLP-1RAs.

## Simple Summary

This study explores how semaglutide, a drug extensively and widely used to treat diabetes and obesity, may cause oral side effects, such as dry mouth, by affecting salivary gland function. Based on the available data, we propose that different GLP-1 receptor agonists activate the salivary gland receptor in distinct ways, triggering pathways that regulate secretion and cell protection. Because semaglutide has strong albumin binding, which leads to prolonged receptor activation, it may disturb the rhythmic calcium and cAMP cross-talk essential for normal salivary secretion. Persistent stimulation may cause receptor desensitization, β-arrestin–mediated internalization, and reduced gland responsiveness. Understanding these mechanisms helps explain why some patients experience oral dryness when taking semaglutide. The findings highlight the importance of monitoring oral health and tailoring treatments to improve comfort and safety for people using semaglutide.

## 1. Introduction

In recent years, the glucagon-like peptide-1 receptor (GLP-1R) and its agonists (GLP-1RAs) have become central to medical research and treatment strategies. The GLP-1RAs, particularly semaglutide, are now used on a global scale for diabetes and obesity, and emerging pharmacovigilance reports suggest that some patients may experience oral and salivary symptoms during therapy, including xerostomia, dysgeusia, oral hypoesthesia, and throat discomfort [1]. Despite their increasing clinical relevance, these symptoms are poorly characterized, and no mechanistic framework currently explains how GLP-1R activation could influence salivary gland function. This gap is striking because salivary flow is essential for oral health, digestion, mucosal protection, and dental homeostasis, and treatment-related hypofunction has measurable consequences for quality of life. GLP-1R belongs to the family of G-protein-coupled receptors (GPCRs), which play a crucial role in transmitting signals across cell membranes. This receptor is widely expressed in many cell types throughout the body, making it an important player not only in metabolism but also in cardiovascular, neural, and skeletal health. The receptor binds specifically to GLP-1, a peptide hormone that regulates blood glucose and lipid metabolism. Through this interaction, GLP-1R helps maintain glucose balance, influences appetite, and supports insulin function. The therapeutic potential of GLP-1R and its agonists has reshaped approaches to managing type 2 diabetes mellitus (T2DM), cardiovascular diseases, obesity, and even neurodegenerative conditions. Following the 2005 Food and Drug Administration approval of exenatide for glycemic control in T2D, several glucagon-like peptide-1 receptor agonists (GLP-1RAs), such as liraglutide, dulaglutide, and semaglutide, have been introduced. These medications have significantly contributed to the management of glucose levels in patients with T2D. Furthermore, at higher doses, liraglutide and semaglutide received approval for weight management in 2014 and 2021, respectively, marking a significant milestone in establishing the role of GLP-1RAs in the treatment of T2D and obesity [1,2,3,4,5]. The GLP-1RAs exert their metabolic effects through the glucagon-like peptide-1 receptor (GLP-1R), a class B GPCR that activates Gs/cAMP, Ca^2+^, and β-arrestin–dependent pathways [3,4,5,6]. Although GLP-1R expression is highest in pancreatic β-cells, transcriptomic datasets indicate that the receptor is present in other tissues, including salivary glands, providing a biologically plausible substrate through which GLP-1R stimulation might influence secretory physiology and that mechanistic basis is needed to contextualize these emerging clinical observations. The aim of this narrative review is to integrate evidence from transcriptomics, GPCR signaling, GLP-1RA pharmacokinetics, β-arrestin biology, and pharmacovigilance data to identify critical gaps and to propose a hypothesis-driven framework for semaglutide-associated oral and salivary dysfunction. 

## 2. Method

A literature search was performed in PubMed/MEDLINE, Scopus, Web of Science, and Google Scholar during March 2025–September 2025, using combinations of the following terms: “GLP-1 receptor”, “GLP-1R agonist”, “GLP-1 signalling”, “semaglutide”, “liraglutide”, “exenatide”, “dulaglutide”, “salivary glands”, “xerostomia”, “hyposalivation”, “oral adverse effects”, “biased agonism”, “β-arrestin”, “cAMP”, “GPCR signaling”, “albumin binding”, “pharmacovigilance”. Because this is a mechanistically oriented narrative review, we focused on biological plausibility, mechanistic evidence, and clinically observed oral effects. Thus, studies were eligible if they met one or more of the following criteria: experimental or translational studies on GLP-1 receptor expression, signaling, trafficking, β-arrestin dynamics, or cAMP–Ca^2+^ cross-talk; preclinical or clinical studies evaluating semaglutide pharmacology, albumin binding, or biased agonism; clinical trials, case reports, observational studies, or FAERS analyses describing oral or salivary adverse effects associated with GLP-1 receptor agonists. The exclusion criteria were: articles not available in English or studies focused solely on metabolic outcomes without relevance to signaling, salivary glands, or oral adverse effects. Two authors independently performed initial screening by title and abstract, followed by full-text review based on the eligibility criteria. Disagreements were resolved by discussion. A total of seventy-eight articles were included in the final synthesis, organized into mechanistic domains: GLP-1R expression in salivary glands; cAMP/PKA signaling and Ca^2+^ regulation; β-arrestin–dependent receptor adaptations; Albumin binding and pharmacokinetic prolongation, and Clinical and pharmacovigilance evidence of oral adverse effects.

## 3. Results of the Literature Review

A total of 183 records were screened, and 76 studies were included in the final synthesis. The data included in this review consisted of transcriptomic datasets (HPA, GTEx, FANTOM5) showing salivary RNA-level expression, mechanistic signaling studies drawn from pancreatic and heterologous cell systems, pharmacokinetic evidence and pharmacovigilance data and clinical case series. Collectively, these references support indirect mechanistic plausibility, but no direct experimental confirmation of GLP-1R function in human salivary glands was found. This striking evidence gap underscores the need for mechanistic and controlled clinical studies to clarify whether semaglutide uniquely affects salivary secretion. By synthesizing transcriptomic, pharmacokinetic, signaling, and pharmacovigilance findings into a coherent mechanistic framework, this narrative review addresses an unrecognized area of GLP-1 biology and provides the first integrated hypothesis on how prolonged GLP-1R activation could disrupt salivary gland function. Given the global rise in GLP-1RA prescriptions and the impact of xerostomia on oral health, nutrition, and quality of life, defining these mechanisms is clinically urgent. Our work therefore establishes a foundation for future translational studies and highlights an overlooked domain of GLP-1RA safety that has direct relevance for dentistry, endocrinology, and patient care.

## 4. Discussion

The findings of this narrative review demonstrate that while GLP-1R is detectable in human salivary glands and multiple mechanistic pathways are well characterized in other tissues, no direct experimental studies have examined GLP-1R signaling, trafficking, or functional outcomes in salivary glands. Therefore, in the interpretation of semaglutide-related oral adverse effects we integrated indirect evidence—from transcriptomic datasets, pancreatic and GPCR signaling models, β-arrestin biology, and pharmacokinetic behavior. In the following sections, we synthesize these mechanistic domains to articulate a biologically plausible framework for semaglutide-associated salivary hypofunction.

### 4.1. GLP-1: Origin and Function

GLP-1 is a peptide hormone derived from the breakdown of proglucagon, a larger precursor molecule. It is produced in three main locations: enteroendocrine L-cells of the intestinal mucosa, pancreatic α-cells, and certain neurons within the nucleus of the solitary tract in the brainstem. After food intake, intestinal L-cells release GLP-1 into circulation in response to nutrient and neuroendocrine signals. The hormone is produced as different molecular variants, including GLP-1(8-36) amide and GLP-1(8-37). Regardless of the form, its primary role is as an incretin hormone—a regulator of postprandial (after meal) blood glucose levels. GLP-1 stimulates insulin secretion in a glucose-dependent manner, suppresses glucagon release, slows gastric emptying, and promotes satiety by acting on appetite centers in the brain. However, its activity in the bloodstream is short-lived. Natural GLP-1 is rapidly degraded within one to two minutes by the enzyme dipeptidyl peptidase-4 (DPP-4), which sharply limits its therapeutic usefulness.

### 4.2. GLP-1 Receptor (GLP-1R) in Salivary Glands

The GLP-1 receptor is a GPCR that responds specifically to GLP-1. While it is highly expressed in pancreatic β-cells, its distribution extends far beyond the pancreas. GLP-1R can be found in the heart, kidneys, lungs, liver, and central nervous system and gastrointestinal tract as well as in salivary glands [7]. Based on the Human Protein Atlas (HPA) page for GLP1R in salivary gland, RNA expression derives from three transcriptomic datasets: HPA bulk RNA-seq, GTEx RNA-seq, and FANTOM5 CAGE. The HPA dataset includes three normal salivary-gland tissue samples (ages 21–68; nTPM 1.3–3.2), while the GTEx minor salivary-gland panel includes 181 donors (mean 0.2 nTPM, range 0.0–1.3). Both HPA and GTEx report expression in normalized transcripts per million (nTPM), whereas FANTOM5 provides scaled CAGE tags per million (sample counts not specified on the GLP1R gene page). HPA assigns GLP1R to RNA expression cluster 8 with a cluster confidence of 1.0; however, it is worth noting that normal-tissue protein annotation is pending, meaning RNA data do not confirm protein presence or function. All dataset values and sample-level information are available via the GLP1R salivary-gland RNA page: https://www.proteinatlas.org/ENSG00000112164-GLP1R/tissue/Salivary+gland#rnaseq (Accessed on 15 November 2025). Thus, GLP1R RNA is expressed at low to moderate levels in salivary glands with cellular distribution mainly in glandular and ductal cells, with variable expression across donors. Expression is biologically relevant but not abundant—consistent with the idea that GLP1R plays a modulatory rather than dominant role in salivary gland function (Table 1).

### 4.3. GLP-1 Receptor Agonists (GLP-1RAs)

Because natural GLP-1 is unstable, GLP-1RAs result from intricate structural modifications to GLP-1, enabling them to not only replicate the pharmacological functions of GLP-1 but also impede its hydrolysis by DPP-4, thereby extending the drug’s half-life [8,9]. Structural modifications allow these drugs to remain active longer in circulation, producing a stronger and more sustained biological effect [10]. Currently available GLP-1RAs vary in their molecular structure and dosing schedule. Some require daily injections, while others are long-acting and administered only once weekly. Newer formulations aim to improve patient convenience further, including orally available tablets. These innovations improve adherence and broaden therapeutic use. Thus, different farmacocinetic profiles associate with different adverse effects [1,11]. The report frequency of gastrointestinal disorders related to semaglutide was significantly higher than that of non-semaglutide in the overall database with the ROR of 4.21. The 45 semaglutide-related gastrointestinal AEs also showed statistically significant signal strengths as compared to non-semaglutide-associated gastrointestinal AEs, with values of signals ranging from a ROR025 of 1.01 (hypoaesthesia oral) to 42.03 (eructation). The association between semaglutide and gastrointestinal disorders remained when stratified by age, body weight, sex and reporter type. Among the 45 gastrointestinal AEs, 17 new and unexpected AEs, including dry mouth, hypophagia, bowel movement irregularity, etc., were detected in our pharmacovigilance study, which were not reported in the drug label (Table 2). The exact effects of semaglutide on these AEs and the mechanisms of this potential association were not completely explored, requiring further clinical investigation [1].

Based on analysis of The Food and Drug Administration’s (FDA) Adverse Effect Reporting System (FAERS), adverse effects of GLP-1 RA-related medications including semaglutide, exenatide, liraglutide, dulaglutide, and tirzepatide were analyzed represent 47 cumulative years of observation across all drugs included in the analysis [11]. Semaglutide was also the only drug to display significant ROR for throat tightness (1.69) and throat irritation. Dysgeusia had significant ROR and PRR in exenatide (ROR 4.02:PRR 4.00), liraglutide (ROR 3.88; PRR 3.86), and semaglutide (ROR 3.67; PRR 3.66). Dry mouth had significant ROR signals in exenatide (1.26), liraglutide (1.80), and semaglutide (3.21) with only semaglutide having a significant PRR (3.19). However, spontaneous reporting systems are subject to substantial limitations, including under-reporting, confounding by indication (e.g., diabetes-related xerostomia), channeling bias toward older or more comorbid patients, and polypharmacy involving xerostomia-inducing drugs such as antidepressants and anticholinergics. Therefore, the observed ROR/PRR elevations indicate signal strength rather than incidence or causal relation. Recent case series has shown 3 cases for patients who were using semaglutide and developed severe secondary hyposalivation [12]. One interesting observation in this report is the onset of hyposalivation in which it was noted 4 weeks after starting semaglutide. The known mechanism of action and body water loss properties for semaglutide may explain the risk of hyposalivation in patients on semaglutide. However, water intake was within the range of normal and considering the severity and duration of reported symptoms, dehydration was less likely to be the underlying cause. In addition, no history of radiation therapy to the head and neck area, was reported by either patient. Another differential diagnosis considered was systemic disease-related hyposalivation. Although dehydration, prior head-and-neck irradiation, and systemic disease were considered and judged unlikely, the sample size is too small to infer generalizable risk or mechanism. 

### 4.4. GLP-1 Signaling Pathway

Glucagon-like peptide-1 (GLP-1) exerts its physiological effects by binding to the GLP-1 receptor (GLP-1R), a class B1 G-protein-coupled receptor (GPCR). [13,14] abundant in pancreas, but also present in salivary glands. In pancreas, upon activation, GLP-1R couples to heterotrimeric G-proteins, primarily stimulating adenylyl cyclase to increase intracellular cyclic AMP (cAMP). Elevated cAMP activates protein kinase A (PKA), which enhances glucose-dependent insulin gene expression and secretion while suppressing glucagon release [15,16]. In parallel, cAMP activates the exchange protein directly activated by cAMP (EPAC), which regulates vesicle priming and exocytosis, thereby fine-tuning insulin release [17,18,19]. At the transcriptional level, cAMP activates the cAMP response element-binding protein (CREB), which regulates insulin gene expression [20,21,22]. Beyond cAMP-mediated mechanisms, GLP-1 signaling also engages the phosphoinositide 3-kinase (PI3K)/protein kinase B (Akt) pathway, which is critical for preserving the survival, proliferation, and function of pancreatic β-cells [23,24,25,26]. Following GLP-1R activation, the βγ subunits of GPCRs directly activate class I PI3Ks, especially the α and β isoforms [27]. These enzymes consist of regulatory and catalytic subunits, which may interact directly with the receptor or indirectly via adaptor proteins such as insulin receptor substrate (IRS) [28,29,30]. PI3K activation leads to the phosphorylation of phosphatidylinositol-4,5-bisphosphate (PIP2), generating phosphatidylinositol-3,4,5-trisphosphate (PIP3) [31]. PIP3 serves as a docking site for Akt (protein kinase B), enabling its recruitment and phosphorylation [32]. The PI3K/Akt pathway remains essential in this context, as it ensures β-cell survival, proliferation, and effective glucose regulation [33,34]. Their activity is fine-tuned by GPCR kinases (GRKs), which phosphorylate the receptor and enable β-arrestin binding. β-arrestins were first recognized for desensitizing GPCRs by uncoupling them from G-proteins and promoting receptor internalization via clathrin-coated pits, but are now known to act as signaling scaffolds themselves, initiating distinct pathways like ERK, JNK, or NF-κB activation. This dual role underpins the concept of biased agonism, where different ligands selectively favor either G-protein-mediated or β-arrestin-mediated signaling, allowing for fine control over therapeutic outcomes and side-effect profiles. Arrestins act as scaffolds to promote recruitment and activation of mitogen-activated protein kinases (MAPKs) that are linked to protection against apoptosis and pancreatic beta-cell survival and growth [35]. GLP-1R-mediated recruitment of arrestins also leads to transactivation of epidermal growth factor receptor, activation of phosphatidylinositol 3-kinase and downstream nuclear translocation of protein kinase Cζ to promote beta cell proliferation, insulin gene expression, and insulin synthesis and secretion [27,36].

Salivary glands, however, rely on a similar set of GPCR-driven cascades to regulate secretion [37]:

- Parasympathetic M3 muscarinic receptors (Gq-coupled) activate phospholipase C → IP3-mediated Ca^2+^ release → fluid (aqueous) secretion.
- Sympathetic β-adrenergic receptors (Gs-coupled) activate adenylyl cyclase → increase cAMP → PKA-dependent pathways that regulate protein-rich exocytotic secretion (e.g., amylase, mucins).

Thus, in salivary acinar cells, cAMP/PKA/EPAC signaling plays a role analogous to pancreatic insulin release, but here it fine-tunes exocytosis of salivary proteins and enzymes rather than insulin granules. Thus, since transcriptomic atlases (HPA, GTEx, FANTOM5) suggest that GLP-1R is expressed in salivary glands, when activated, GLP-1R would engage the same cAMP–PKA–EPAC and PI3K–Akt modules, potentially influencing [38,39,40]:

- Vesicle priming and exocytosis of salivary proteins (via cAMP signalling).
- Cellular protection and survival of acinar and ductal cells (via EGF).
- CREB-dependent transcription, possibly altering the synthesis of secretory proteins or ion transporters that regulate saliva composition.

### 4.5. Biased Agonism of GLP-1R

Biased agonism describes the ability of different ligands to stabilize distinct receptor conformations, preferentially activating some pathways (e.g., G protein–mediated cAMP signaling) while avoiding others (e.g., β-arrestin recruitment), which allows for fine-tuning pharmacological outcomes [41,42]. Unlike most GPCRs that internalize through a canonical GRK–β-arrestin–clathrin mechanism [43], GLP-1R displays atypical trafficking properties. GLP-1R internalizes via an arrestin-independent, GRK-dependent, clathrin- or caveolae-mediated pathway [44]. After endocytosis, GLP-1R continues to signal from early endosomes, producing sustained cAMP signaling by internalized GLP-1Rs, but without increasing insulin release [45]. Acute β-arrestin-1 engagement can facilitate insulin release, but prolonged or excessive recruitment may reduce insulinotropic efficiency by altering receptor recycling and signaling balance [46]. Experimental evidence from β-arrestin2 knockout models shows reduced GLP-1R trafficking toward lysosomes and increased routing toward the trans-Golgi network, thereby altering receptor recycling and degradation dynamics [47].

Different GLP-1 receptor agonists (GLP-1RAs) show distinct patterns of receptor handling. For example, semaglutide and native GLP-1 promote relatively conventional recycling and internalization, whereas biased agonists like tirzepatide favor sustained receptor retention at the plasma membrane with reduced cytosolic trafficking [48]. These divergent patterns of β-arrestin utilization and endosomal signaling may underlie differences in drug-specific clinical profiles, including glucose control, durability of weight loss, and gastrointestinal tolerability.

The study of Dawed and colleagues investigated whether genetic variants could predict response to GLP-1 receptor agonists in 4571 adults with type 2 diabetes across observational studies and RCTs, measuring HbA1c change after six months [49]. The findings underscore that GLP-1 response is a polygenic trait influenced by both common and rare loci, and that integrating pharmacogenomics with functional analyses may advance precision medicine in type 2 diabetes. Namely, they found that the common GLP1R variant Gly168Ser (rs6923761) was associated with poorer response, while rare variants in ARRB1 (notably Thr370Met) enhanced response by reducing GLP1R internalisation and boosting cAMP and insulin secretion, with stronger effects in Hispanic and American Indian/Alaska Native populations. A combined genetic score showed up to a 30% difference in HbA1c reduction be-tween low- and high-response carriers, highlighting the clinical relevance of these variants, though effects were specific to GLP-1 receptor agonists and not other drug classes.

### 4.6. Semaglutide as a “Not Clinically Confirmed as Biased” Agonist

Semaglutide, is a drug with 94% homology with human GLP-1. Its chemical structure consists of 31 amino acids with two amino acid substitutions (Aib8 and Arg34) at position 8 reduces susceptibility to degradation by dipeptidyl peptidase-4 (DPP-4). Studies such as SUSTAIN, PIONEER, and STEP highlight its superiority compared to other GLP-1 receptor agonists and anti-obesity drugs in [50]. Moreover, the modifications improve the specific high-affinity binding to albumin, which slows down the degradation of semaglutide in plasma and results in decreased renal clearance and in prolongation of the half-life of semaglutide to ~1 week, making it appropriate for once-weekly administration [51]. As capable of β-arrestin recruitment, semaglutide can trigger receptor desensitization and internalization, which may limit the duration of signaling in β-cells [52]. Thus, semaglutide shows reduced β-arrestin recruitment and receptor internalization compared to endogenous GLP-1; however, current evidence does not conclusively classify semaglutide as a functionally or clinically biased GLP-1R agonist [52]. In comparative studies, complete suppression of β-arrestin recruitment produced slower internalization, reduced downregulation, and longer-lasting cAMP signaling than semaglutide [52], indicating that semaglutide retains functionally meaningful arrestin engagement, even if not at the highest level among GLP-1R agonists. Thus, semaglutide still supports measurable GLP-1R internalization under physiological albumin conditions, leading to receptor loss from the surface which could limit the duration of its glucose-lowering effects compared with “fully biased” ligands. In line with this, the complete abolition of β-arrestin signaling prolonged anti-hyperglycemic effects (superior glucose tolerance after 72 h compared to semaglutide) and enhanced chronic weight loss in mice [52].

The study of Sonoda et al. [46] through a combination of coimmunoprecipitation, RNAi knockdown, immunoblotting, cAMP assays, and pharmacological assays demonstrated that β-Arrestin-1 knockdown broadly attenuated GLP-1 signaling, causing decreased ERK and CREB activation and IRS-2 expression as well as reduced cAMP levels and impaired insulin secretion. However, β-arrestin-1 knockdown did not affect GLP-1 R surface expression and ligand-induced GLP-1 R internalization/desensitization. Thus, β-arrestin-1 interacts directly with GLP-1R and amplifies GLP-1 signaling and may influence β-cell performance and growth.

On the other hand, study of Bitsi et al. in vivo showed that β-arrestin 2 normally promotes receptor degradation and limits sustained signaling [47]. Namely, glycemic responses to the pharmacological GLP-1R agonist exendin-4 in adult β cell–specific β-arrestin 2 knockout (KO) mice showed that when β-arrestin 2 is absent, acute responses are impaired, but prolonged responses are enhanced through reduced receptor ubiquitination, avoidance of lysosomal degradation, and redirection of active receptors toward the trans-Golgi network, where intracellular signaling is sustained [47]. Of note, the role as loss of β-arrestin-1 does not affect GLP-1R internalization on the other hand, enhancing β-arrestin-2 action by the overexpression of G protein receptor kinase 5 (GRK5) increases GLP-1R endocytosis [53]. Nevertheless, these data establish that biased agonism at the GLP-1R is a key determinant of functional outcomes. Agonists that preferentially engage G protein signaling while avoiding β-arrestin recruitment (such as exendin-phe1) produce stronger, longer-lasting insulin release. Conversely, agonists that drive β-arrestin-2 engagement promote receptor endocytosis and desensitization, reducing prolonged secretory capacity. cAMP responses to exendin-4, with an intermediate pattern, were potentiated in arrestin-null cells, showing both increased potency and a trend toward higher efficacy over time compared to wild-type. This indicates that β-arrestins act primarily as inhibitory on GLP-1R cAMP signaling during chronic agonist exposure, rather than being essential for receptor internalization [53].

**Figure 1:**
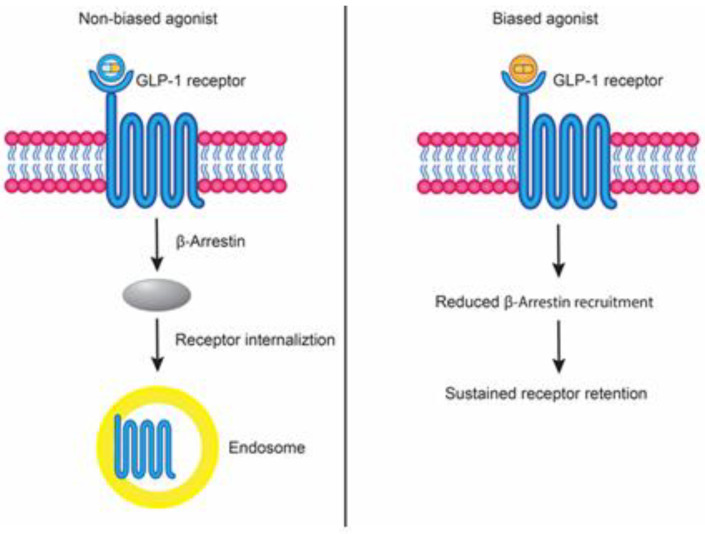
Comparison of Biased and Non-biased GLP-1 Receptor Agonism. Schematic representation of the distinct signaling mechanisms induced by agonists at the GLP-1 receptor. Semaglutide acts as “not clinically confirmed as biased” agonist, promoting β-arrestin recruitment, receptor internalization, and endosomal signaling. In contrast, tirzepatide functions as a biased agonist, exhibiting reduced β-arrestin recruitment and sustained receptor signaling at the cell surface. These mechanistic differences may contribute to their divergent pharmacodynamic profiles and clinical effects.

Semaglutide, similarly, is characterized as “not clinically confirmed as biased” GLP1R agonist [52], which preserve acute signaling support while also enabling desensitization pathways [47], which may contribute to clinical observations such as tachyphylaxis of gastrointestinal side effects. From a therapeutic perspective, semaglutide’s may thus provide sufficient β-arrestin 1 engagement in responses; on the other, it remains vulnerable to β-arrestin 2–driven desensitization over time, which could limit its signaling durability in β cells under chronic exposure. This mechanistic framework may help explain why semaglutide shows robust efficacy for glycemic control and weight loss, but also why some adverse effects and tissue-adaptive responses emerge with continued administration (Figure 1).

### 4.7. β-Arrestins in Receptor Signaling and Adaptation

β-arrestins are versatile regulators of GPCR behavior, contributing both to signal termination and to alternative signaling and adaptive receptor responses.

#### 4.7.1. Desensitization and Receptor Trafficking

Most agonist-activated GPCRs undergo rapid desensitization through a coordinated two-step process: first, phosphorylation of the receptor by GRKs, and second, recruitment of β-arrestins to these phosphorylated sites, which sterically block further G-protein coupling [54]. This ensures that signaling via G-proteins is reduced once receptors are adequately stimulated. However, β-arrestins play a far more complex role than mere inhibition. By functioning as scaffolding proteins, they recruit downstream effectors that degrade second messengers and fine-tune the signaling output. For instance, β-arrestin-2 recruits PDE4D3 and PDE4D5 to the β_2_-adrenergic receptor (β_2_AR), reducing cAMP levels and PKA activity [55]. 

#### 4.7.2. β-Arrestin-Dependent Signaling and Functional Duality

The recognition that β-arrestins not only terminate signaling but also propagate it has led to the concept of bimodal GPCR signaling [43], consisting of G-protein-dependent and β-arrestin-dependent pathways. β-arrestins have been shown to function as critical adaptors for agonist-induced endocytosis of GPCRs [56,57], and as important adaptors for agonist-induced ubiquitination of GPCRs [58,59], and scaffold MAPK cascades such as ERK1/2 and JNK3 within endosomes [60]. This scaffolding allows GPCRs to maintain signaling even when G-protein activity is disabled [61]. Thus, a receptor can continue to transmit signals solely via β-arrestins, and ligands can selectively bias signaling toward either G-proteins or β-arrestins [62].

Paradoxically, β-arrestins can also prolong G-protein signaling instead of blocking it in a subset of receptors. This counterintuitive effect arises from the conformational plasticity of β-arrestins: certain conformations inhibit G-protein coupling, while others allow for persistent G-protein activity despite arrestin binding. Such behavior has been observed in “class B” GPCRs, including PTH1R and GLP-1R, which form stable β-arrestin complexes and sustain both prolonged G-protein and ERK signaling from endosomes [63,64]. By contrast, “class A” receptors like the β_2_AR preferentially form transient complexes with β-arrestin-2, leading to rapid termination of signaling and receptor recycling [65].

In the context of the GLP-1R, β-arrestin-2 is the predominant isoform and has been shown to mediate a dual role in receptor adaptation. Acutely, β-arrestin-2 supports signaling by stabilizing high-affinity receptor conformations, facilitating cAMP generation, calcium responses, and insulin secretion in pancreatic β-cells. However, during prolonged stimulation, β-arrestin-2 exerts a desensitizing influence, promoting receptor down-regulation and lysosomal targeting, thereby reducing long-term responsiveness. In contrast, β-arrestin-1 can partially compensate when β-arrestin-2 is absent, but its recruitment is linked to stronger engagement of phosphodiesterase-4 (PDE4), leading to enhanced cAMP degradation and acute signaling defects. This isoform-specific interplay underscores that receptor adaptation is not a linear process but a dynamic balance between acute signal support and chronic desensitization [46,47,52]. Altogether, these findings establish β-arrestins as multifunctional regulators of receptor signaling and adaptation. They terminate GPCR signaling through desensitization, orchestrate receptor trafficking, scaffold diverse signaling complexes, and in some cases sustain G protein activity. Their ability to dictate biased signaling outcomes makes them central to GPCR pharmacology and valuable targets for drug discovery aiming at pathway-selective therapies.

### 4.8. Albumin Binding as a Pharmacokinetic Determinant of Semaglutide Effects

**Figure 2:**
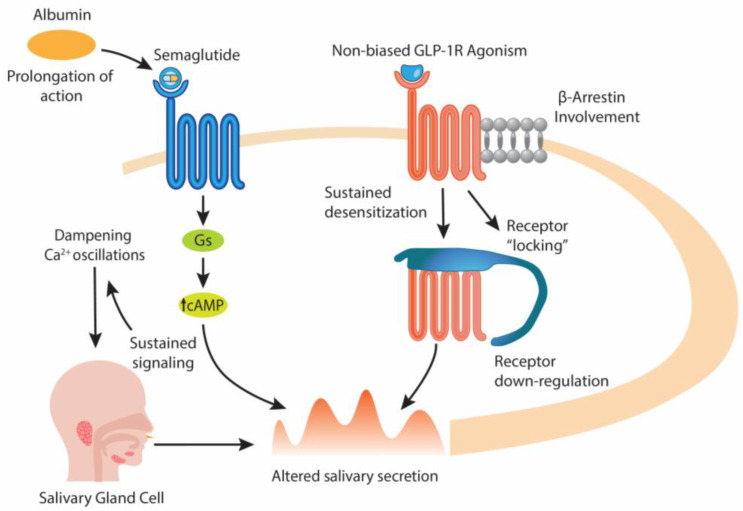
Mechanistic illustration of semaglutide’s “not clinically confirmed as biased” GLP-1 receptor agonism and its potential impact on salivary gland signaling. Semaglutide engages the GLP-1 receptor and activates both Gs-mediated cAMP signaling and β-arrestin–dependent pathways, meaning that neither pathway is preferentially or selectively favored based on currently available evidence. Persistent systemic exposure is prolonged by high-affinity albumin binding, which limits renal clearance and maintains circulating drug concentrations, leading to sustained receptor occupancy. Following receptor activation, elevated intracellular cAMP may interact with Ca^2+^-dependent oscillatory signaling, which is essential for physiological, rhythmic salivary secretion. Concurrent β-arrestin recruitment can promote receptor internalization and desensitization, potentially reducing receptor responsiveness over time. Together, these processes represent a hypothesis-driven framework through which prolonged GLP-1R activation may modulate salivary gland cell signaling dynamics and influence secretory output, although direct human salivary-gland pharmacodynamic data are not yet available.

Semaglutide contains a large hydrophobic fatty diacid side chain that anchors the peptide to human serum albumin [66]. Albumin is the most abundant plasma protein, circulating at concentrations of 35–50 g/L with a half-life of approximately 19 days. It serves as a natural carrier for fatty acids, hormones, and drugs through multiple binding sites and undergoes neonatal Fc receptor-mediated recycling, which protects it from renal clearance and lysosomal degradation. Drugs engineered to bind albumin therefore benefit from prolonged systemic exposure. For semaglutide, this strategy results in a pharmacokinetic half-life of approximately seven days, enabling once-weekly dosing [6]. Due to albumin binding, there is a continuous reservoir of semaglutide in plasma, which leads to persistent exposure of GLP-1 receptors in pancreas as well as in extra-pancreatic targets (hypothalamus, liver, salivary glands). This prolonged exposure ensures continuous activation of GLP-1R, improving fasting and postprandial glucose control compared to short-acting agonists (like exenatide) [67]; however, in exocrine tissues such as salivary glands, continuous receptor engagement may impair physiological oscillations of signaling. Salivary secretion depends on finely tuned receptor activity, and sustained activation may paradoxically reduce glandular responsiveness, contributing to the salivary hypofunction. In contrast, shorter-acting GLP-1RAs such as exenatide are rapidly cleared and do not maintain chronic receptor occupation. Liraglutide, although also albumin-associated via fatty acid acylation, displays weaker binding and a shorter half-life (~13 h) than semaglutide [6]. Thus, semaglutide’s unique depth of albumin association could explain its stronger tendency to induce tissue-level adaptations, including those leading to dry mouth. Importantly, sustained receptor occupation due to albumin binding may magnify the functional consequences of even modest β-arrestin recruitment (Figure 2). Thus, due to its strong albumin binding and prolonged systemic exposure, semaglutide may maintain longer receptor occupancy, which could theoretically influence receptor adaptation dynamics in tissues expressing GLP-1R, including salivary glands; however, this remains hypothesis-generating as no direct salivary pharmacodynamic measurements have been performed to date.

### 4.9. Clinical Correlates: Oral Adverse Effects

Real-world pharmacovigilance studies suggest that semaglutide is disproportionately associated with gastrointestinal adverse events compared to other GLP-1Ras [10,11]. Noteworthy, due to the multifactorial nature of xerostomia, any association between semaglutide and reduced salivary function must be interpreted with caution until controlled studies accounting for metabolic, autoimmune, neurological, pharmacologic, and age-related confounders are available. Nevertheless, while nausea, vomiting, and diarrhea dominate as symptoms in the reports, oral adverse events such as hypoaesthesia and xerostomia are increasingly recognized especially with semaglutide [1,10,11]. The median time-to-onset of gastrointestinal events is approximately four weeks [12], consistent with the early phase of receptor adaptation. Given that salivary glands express GLP-1R at low but detectable levels, chronic overstimulation could plausibly extend these adverse patterns to oral secretory dysfunction.

Comparative analyses suggest that liraglutide, exenatide, and dulaglutide display different balances of albumin binding and GLP1 receptor signaling bias, and thus may pose lower xerostomia risk. For example, exenatide, with rapid clearance, minimizes prolonged receptor overstimulation, while liraglutide’s weaker albumin binding and shorter half-life limit its potential for sustained β-arrestin–driven desensitization. This highlights semaglutide’s unique pharmacological niche: superior efficacy accompanied by variable side effects.

Salivary secretion often requires rhythmic, pulsatile activation of secretory apparatus, for example, bursts of saliva release in response to food stimuli. The key event in activation of fluid secretion is an increase in [Ca^2+^]i triggered by inositol 1,4,5-trisphosphate (IP3)-induced release of Ca^2+^ from ER via the IP3 receptor (IP3R) and cells finely tune the generation and amplification of [Ca^2+^]i signals for regulation of cell function [68]. These pulsatile Ca^2+^ signals depend on the ability of receptors to repeatedly activate and reset, and allow acini to engage in electrolyte transport, fluid secretion, and protein content in a balanced manner [69].

Chronic exposure to GLP-1 receptor agonists (GLP-1RAs) such as semaglutide, due to albumin binding, and recruitment of β-arrestins may disturb this rhythm. Sustained stimulation stabilize β-arrestin binding, “locking” the receptor in an internalized state [70], which promotes receptor ubiquitination, and receptor down-regulation as shown with muscarinic receptor agonists [71]. The outcome thus, may be the long-term desensitization and reduced responsiveness of salivary acinar cells. On the other side, sustained GLP1R/cAMP signaling could dampen or alter the normal Ca^2+^ oscillation pattern via Ca^2+^ -cAMP cross-talk [72]. Thus, either sustained cAMP signaling or receptor desensitization could alter salivary secretion.

Namely, salivary secretion in acinar cells is regulated by the interplay of cAMP and calcium signaling pathways. Within this framework, the PKA pathway is central: cAMP activation triggers PKA, which promotes protein secretion, facilitates fluid secretion by elevating intracellular calcium levels, and activates cAMP response element–binding protein (CREB), a key transcription factor coordinating these downstream signaling events [73]. However, while short bursts of cAMP may transiently potentiate Ca^2+^ release and secretion [72], sustained cAMP elevation could suppress oscillatory Ca^2+^-dependent Cl^−^ currents, partly by: redistributing Cl^−^ efflux to non-luminal pathways, reducing the amplitude of oscillatory pulses critical for fluid secretion and, possibly dampening Na^+^-K^+^-2Cl^−^ cotransporter activity [74]. If semaglutide favors sustained Gs/cAMP signaling, cAMP responses may disrupt cross-talk between cAMP and Ca^2+^ pathways as it has been shown that cAMP-elevating agents (e.g., forskolin + IBMX) could suppress Ca^2+^-activated oscillatory Cl^−^ secretion in salivary acinar cells by reducing the amplitude/duration of Ca^2+^-driven pulses [74]. Since Cl^−^ secretion induces fluid output, suppression of oscillatory currents may result in reduced salivary flow. Furthermore, receptor internalization via β-arrestins introduces “pauses” that allow signaling to reset. If semaglutide reduces β-arrestin recruitment, receptors remain at the membrane, sustaining Gs/cAMP signaling and affecting Ca^2+^ oscillations. Over time, this alters the salivary secretion, manifesting clinically as reduced fluid output and xerostomia. 

The risk of salivary dysfunction may vary across GLP-1RAs. Currently approved GLP-1 agonists, including liraglutide, semaglutide, dulaglutide and lixisenatide, as well as exendin-4 have been examined for bias between cAMP and β-arrestin-2 recruitment [75]. The results showed that all were full agonists for both pathways except exendin-4, which showed a subtle reduction in efficacy for β-arrestin-2, while only liraglutide showed statistically significant bias in favour of β-arrestin-2 recruitment over cAMP [75]. Thus, Liraglutide, which recruits β-arrestins more strongly than semaglutide, may allow for more receptor recycling [76], in terms of salivary secretion, and preservation of oscillatory Ca^2+^-driven secretion. As a result, patients on liraglutide might experience fewer xerostomia-related symptoms compared with those on prolonged semaglutide therapy. While the mechanistic framework presented here aligns with established GLP-1R signaling and trafficking paradigms, the proposed Ca^2+^–cAMP disruption and β-arrestin–mediated adaptation in salivary acinar cells should be considered hypothesis-generating, as direct salivary-tissue evidence under semaglutide exposure is currently lacking. Findings from pancreatic β-cell and other GLP-1R–expressing models may not fully recapitulate exocrine gland physiology, and future human or experimental salivary-gland studies are needed to validate these mechanistic possibilities.

## 5. Conclusions

This narrative review demonstrates that semaglutide’s oral adverse effects cannot yet be explained by direct salivary-gland data, but the convergence of three mechanistic domains—albumin-mediated continuous receptor stimulation, bias toward sustained Gs/cAMP signaling, and β-arrestin–dependent desensitization—provides a coherent hypothesis for salivary hypofunction. When contextualized with FAERS disproportionality signals and the case series of severe hyposalivation, these mechanisms suggest that semaglutide may disrupt the rhythmic Ca^2+^–cAMP dynamics required for normal secretion. Thus, while causality cannot be established, the biological plausibility is strong and warrants targeted mechanistic studies. Since xerostomia significantly reduces quality of life and increases the risk of oral infections, mucosal irritation, and dental caries, counseling of patients on semaglutide should include awareness of this potential side effect, alongside supportive strategies such as hydration, saliva substitutes, and enhanced oral hygiene.

## Figures and Tables

**Table 1:** GLP1R RNA and protein expression in human salivary glands.

Source	Method	Average Expression	Range/Details	Interpretation
HPA RNA-seq	RNA sequencing (HPA samples)	~2.1 nTPM	1.3–3.3 nTPM across donors (ages 21–68)	Low–moderate expression, mainly in glandular and ductal cells
GTEx	RNA-seq (minor salivary glands, 162 samples)	~0.1 nTPM	0.0–1.0 nTPM	Very low expression, often near detection limit
FANTOM5 CAGE	CAGE (Cap Analysis of Gene Expression)	1.8–3.4 TPM	Parotid: 1.8; Submandibular: 2.6; Unspecified gland: 3.4	Confirms low–moderate expression in major salivary glands

**Table 2:** Semaglutide characteristics relevant for its clinical effects. “↑” means increased.

Aspect	GLP1R Signalling (General)	Semaglutide Specifics
Principal signaling pathway	Gs → ↑ cAMP → PKA & EPAC2	Strong, sustained cAMP signaling
Complementary signaling pathway	PI3K/Akt, MAPK, β-arrestin	Reduced β-arrestin recruitment and receptor internalization compared to endogenous GLP-1~“not clinically confirmed as biased” agonist
Tissue distribution	Pancreas, brain, heart, kidney, GI, salivary ducts	Same, but sustained exposure may alter receptor availability (e.g., salivary ducts)
Pharmacokinetics	Native GLP-1 t½ ~2 min	Modified for albumin binding → t½~7 days
Receptor dynamics	Pulsatile exposure (short-acting agonists)	Continuous exposure → adaptive downregulation possible
Clinical efficacy	Improves glucose and weight	Greater HbA1c reduction and weight loss
Side effects	GI upset, rare pancreatitis	Increased hypoaesthesia and xerostomia

## Data Availability

No new data were created or analyzed in this study. Data sharing is not applicable to this article.

## Abbreviations

The following abbreviations are used in this manuscript:

GLP-1	glucagon-like peptide-1
GLP-1R	glucagon-like peptide-1 receptor
GLP-1RAs	glucagon-like peptide-1 receptor (GLP-1R) agonists
GPCR	G-protein-coupled receptor
T2DM	type 2 diabetes mellitus
DPP-4	enzyme dipeptidyl peptidase-4
AEs	adverse effects
FDA	Food and Drug Administration’s
FAERS	FDA Adverse Effect Reporting System
PKA	protein kinase A
EPAC	exchange protein directly activated by cAMP
CREB	cAMP response element-binding protein
PI3K	phosphoinositide 3-kinase
Akt	Protein kinase B
IRS	insulin receptor substrate
PIP2	phosphatidylinositol-4,5-bisphosphate
PIP3	phosphatidylinositol-3,4,5-trisphosphate
GRK	GPCR kinase
ERK	extracellular signal-regulated kinase
JNK	c-Jun N-terminal kinase
NF-κB	nuclear factor kappa-light-chain-enhancer of activated B cells
MAPKs	mitogen-activated protein kinases
IP3	Inositol 1,4,5-trisphosphate
HPA	Human protein atlas
GTEx	Genotype-tissue expression project
FANTOM5	Functional Annotation of the Mammalian Genome 5
EGF	Epidermal growth factor
RCTs	Randomized controlled trials
HbA1c	Glycated Hemoglobin A1c
Gly168Ser	Glycine at position 168 is replaced by Serine
ARRB1	β-arrestin 1
RNAi	Ribonucleic acid interference (RNA interference)
IRS-2	Insulin Receptor Substrate 2
KO	Knockout
PDE4D3	Phosphodiesterase 4D, isoform 3
PDE4D5	Phosphodiesterase 4D, isoform 5
β_2_AR	the β_2_-adrenergic receptor
PTH1R	Parathyroid Hormone 1 Receptor
PDE4	Phosphodiesterase 4
FC	Fragment crystallizable region (receptor)
IP3R	IP3 receptor
IBMX	3-Isobutyl-1-methylxanthine
CAGE	Cap analysis of gene expression
nTPM	Normalized transcripts per million
GI	Gastrointestinal
